# Novel Therapeutic Approaches to the Treatment of Chronic Abdominal Visceral Pain

**DOI:** 10.1100/tsw.2006.98

**Published:** 2006-04-18

**Authors:** Franca Patrizi, Steven D. Freedman, Alvaro Pascual-Leone, Felipe Fregni

**Affiliations:** ^1^ Department of Medicine and Neurology, Beth Israel Deaconess Medical Center, Harvard Medical School, Boston, USA; ^2^ Department of Surgical and Gastroenterological Sciences, Policlinico GB Rossi, University of Verona, Italy

**Keywords:** brain stimulation, transcranial magnetic stimulation, chronic pain, visceral pain, treatment

## Abstract

Chronic abdominal visceral pain (CAVP) has a significant clinical impact and represents one of the most frequent and debilitating disorders in the general population. It also leads to a significant economic burden due to workdays lost, reduced productivity, and long-term use of medications with their associated side effects. Despite the availability of several therapeutic options, the management of patients with CAVP is often inadequate, resulting in frustration for both patients and physicians. This may in part be explained by the lack of understanding of the mechanisms underlying chronic pain; in contrast with acute pain in which the pathophysiology is relatively well known and has several satisfactory therapeutic options. Recently, the development of tools for brain investigation, such as functional magnetic resonance imaging, has provided new insights on the pathophysiology of chronic pain. These new data have shown that plastic changes in the central and peripheral nervous system might play an important role in the maintenance of chronic pain. Therefore, approaches aimed at the modulation of the nervous system, rather than the ones interfering with the inflammatory pathways, may be more effective for chronic pain treatment. We propose that noninvasive central nervous system stimulation, with transcranial magnetic stimulation (TMS), might be a novel therapeutic option for CAVP. This paper will present an overview of the pathophysiology and the available therapies for CAVP, focusing on the recent advances in the treatment of this pathology.

## INTRODUCTION

Chronic abdominal visceral pain (CAVP) is one of the most frequent and debilitating disorders in the general population, critically impacting economy and quality of life[1]. Despite the fact that acute pain is a protective mechanism against potential environmental danger, chronic pain has no such protective role.

For this reason, chronic pain must be considered a real disease state in itself. In addition, CAVP is a heterogeneous disorder with a variety of clinical presentations, such as different pain intensity and frequency of relapses[2]. Therefore, it is important to study CAVP independently.

Our understanding of the etiology, pathophysiology, and natural history of chronic abdominal pain has significantly increased in the past decade, in part because of the new tools to investigate the nervous system[3]. For instance, the development of novel, neuroimaging tools has provided new insights into the brain dysfunction associated with CAVP. Although the precise pathophysiology of CAVP is still far from being elucidated, recent data show that this disorder might be associated with a dysregulation at multiple levels of the of so-called "brain-gut axis"[4], involving both the central nervous system (CNS) and the peripheral nervous system (PNS). Even though several studies have been conducted to elucidate the pathophysiology of chronic pain, *abdominal visceral* chronic pain may have different characteristics and be associated with different areas of nervous system activity.

Another important aspect of CAVP is the treatment of this disorder. Although several options are available for the treatment of acute pain, these therapies usually are not effective to alleviate CAVP. This is, most likely, because the mechanisms associated with chronic pain are different from those of acute pain. For instance, chronic pain is associated with brain plastic changes that might perpetuate the sensation of pain (this has been extensively demonstrated in patients with spinal cord injury {see review[5]} and also other types of pain[6]). Because treatments for acute pain, such as nonsteroidal anti-inflammatory drugs (NSAIDs), target specific inflammatory pathways[7], they are not suitable to alleviate chronic pain. It is critical to understand the pathophysiology of CAVP in order to develop effective treatment targets for this disease. In this paper, we will review the neural networks involved in the pathophysiology of chronic visceral pain (encompassing PNS and CNS) as well as the current and new therapeutic approaches for this disease.

## Epidemiology of Chronic Visceral Pain

Although CAVP is commonly seen by primary care physicians and specialists, there are no reliable numbers for the prevalence of CAVP. Epidemiologic studies fail to identify the number of patients with this condition correctly, perhaps because of the lack of clear pathologic features associated with CAVP; thus, the sample population may vary according to the nature and interpretation of the diagnostic criteria. In addition, using the recent criteria for the diagnosis of functional gastrointestinal disorders, the Rome criteria, several studies showed a different prevalence in functional abdominal pain. Recurrent or chronic abdominal pain can begin in childhood, but the majority of patients that seek medical treatment are in the 20- to 50-year range. The exact prevalence of chronic abdominal visceral pain in children is not known. It seems to account for 2–4% of all pediatric office visits[8]. In adults, chronic functional abdominal pain (excluding other causes, such as irritable bowel syndrome [IBS]) occurs in 1.7% of this population, mainly in women[9].

Epidemiological studies of particular conditions associated with chronic abdominal pain, such as IBS, are useful to estimate the extension of CAVP. For instance, the prevalence of IBS that provides an important contribution to functional visceral pain disorders, as estimated by questionnaire studies, is 2.9%, but population-based studies in the U.S. estimate its prevalence between 5 and 25%, being 3–4 times greater in women than men[10]. A French study that investigated 8,221 subjects found that 23.1% of them suffered from recurrent abdominal pain and the prevalence of IBS was 2.6% according to the criteria developed for this study and 2.1 and 1.1% according to Rome I and II diagnostic criteria, respectively[11]. In a community survey study, researchers found that the prevalence of IBS was 5.1, 6.8, 5.1, and 27.6% according to Rome II, Rome I, Rome (1990), and Rome (1989) criteria[12].

IBS can affect people at any age with the prevalence declining with age; approximately 50% of patients report symptoms onset before the age of 35[13]. The precise incidence of IBS is unclear since the majority of people do not consult a physician, but it has been estimated at 1–2% per year[14]. In children, using the Rome II diagnostic criteria for functional gastrointestinal disorders, a recent study found that

36% of the children with diagnosis of recurrent abdominal pain have symptoms consistent with a diagnosis of IBS[15].

Among the functional gastrointestinal disorders, functional dyspepsia is associated with persistent or recurrent pain or discomfort localized in the upper abdomen with no apparent explanation despite appropriate investigations[16]. Epidemiologic survey suggests that 15–20% of the general population in the Western countries experience dyspepsia over the course of 1 year[17]. However, the real incidence of patients with dyspeptic symptoms might be underestimated as only 25% of people with dyspeptic symptoms seek medical care.

Chronic abdominal pain is also the dominant feature of chronic pancreatitis. Its incidence is assessed to be approximately 50–75 cases per 100,000 patients per year. Along the course of this disease, pain is experienced by 95% of patients[18]. Even though a relatively uncommon pathology, chronic pancreatitis has an important clinical and economical impact, since patients need close medical follow-up and intensive medical therapies for debilitating chronic abdominal pain[19]. Indeed, pain in these patients is often resistant to high doses of narcotics, even if infused epidurally.

Finally, in a significant proportion of patients with CAVP, no formal diagnosis can be made. For this population of patients, estimates of prevalence or incidence of this disease are not available.

## Mechanisms of Abdominal Visceral Pain

Pain is recognized as a multidimensional experience including several coexisting components. The sensory-discriminative component represents the ability to localize a stimulus in space and time and assess its intensity. The affective-motivational component refers to the experience of the unpleasant and emotional aspects of the pain. Finally, the cognitive-evaluative component consists of evaluation and interpretation of the meaning of pain experience[20].

Characteristically, even though visceral pain may be the response to adequate stimuli as distension or inflammation, the severity of pain does not always reflect the severity of the condition causing the pain. Indeed, severe abdominal pain can be associated with even innocuous stimuli such as gas or food passage (hyperalgesia/allodynia) when acting on inflamed or otherwise normal tissue, as in IBS.

Whereas the acute somatic pain pathways have been long identified and well studied, the several components acting in functional chronic visceral pain are a challenging subject. Peripheral visceral receptors, spinal afferent and ascending pathways, and brain subcortical and cortical structures are all possible sites for the elaboration and integration of painful stimuli; thus, mechanisms acting at these levels play a critical role in the pathophysiology of chronic pain and will be further discussed (see Fig. 1).

## Visceral Receptors

The primary afferent neurons in the intestinal wall, in most of the cases, are associated with the initial triggering of pain. These receptors have particular characteristics that render them an important cause of chronic pain; in addition, plastic processes in these receptors can be responsible to sustain chronic pain.

Sensory innervation of the gastrointestinal tract involves all layers of the viscus (mucosa, muscularis, and serosa) with visceral receptors exhibiting different sensitivities. Three distinct classes of visceral receptors have been revealed through electrophysiological studies in experimental investigations: intensity-encoding mechanoreceptors, high-threshold mechanoreceptors, and mechanically insensitive receptors[21]. The intensity-encoding mechanoreceptors have a low threshold to mechanical stimulation, however, they only elicit a painful response if the stimulus intensity or duration increase (e.g., fullness, urge to void)[22,23]. High-threshold mechanoreceptors have a high threshold to mechanical stimuli (around the threshold for pain in humans) and an encoding function contained entirely within the noxious range of stimulation. Finally, mechanically insensitive or "silent" nociceptors acquire spontaneous activity only after tissue insult. This class of receptor may be particularly important in viscera, where chemical

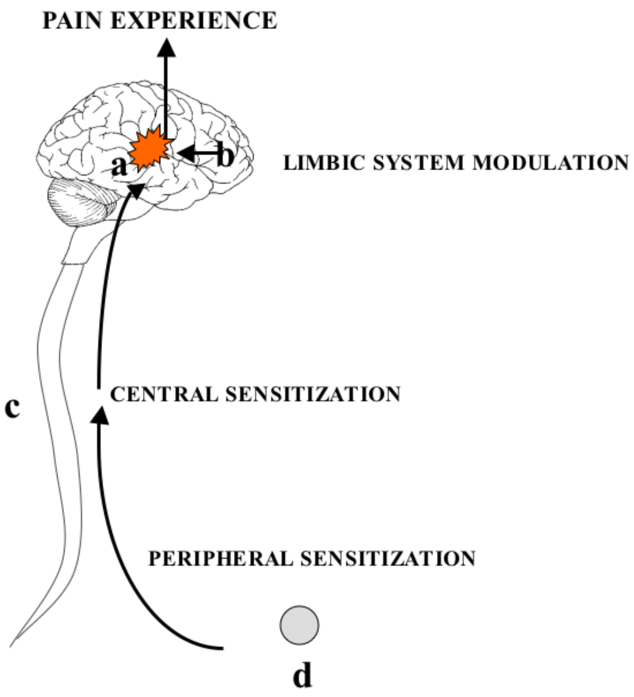

**FIGURE 1.** Diagram showing the pathway of pain from the peripheral receptors to cortical areas (a, secondary somatosensory area; b, limbic areas including the cingulate cortex; c, spinal cord; d, peripheral receptors).

stimuli and inflammation are often much more effective at producing pain than mechanical stimuli. A particularly clear example of this is the heart, where mechanical stimuli do not evoke any sensation, but chemical stimuli and especially ischemia of the cardiac muscle produces intense pain[22]. Therefore, this class of receptors might be important in the pathophysiology of chronic pain as once they are stimulated by tissue injury and inflammation, they may become sensitive to other type of stimuli and be responsible for the maintenance of chronic pain. This process is called sensitization.

Sensitization generally occurs after inflammation and indicates an increase in response magnitude, sometimes accompanied by an increase in spontaneous activity and/or a decrease in response threshold. Sensitization of cutaneous nociceptors has long been recognized as an initial, important event to the development of cutaneous hyperalgesia. Mechanosensitive visceral afferent fibers, both those with low- and high-response thresholds, have the ability to sensitize and thus contribute to altered sensations arising from the viscera.

Several chemical mediators derived from immune and nonimmune cells (bradykinin, serotonin, histamine, prostaglandin $E_2$, KCl, and nerve growth factor [NGF]) have been proposed[24,25] as contributors to acute sensitization of mechanoreceptors or to awakening of silent nociceptors[26]. Importantly, acute sensitization often is followed by long-term changes in the PNS and CNS activity that can maintain visceral hyperalgesia. As proposed by Gebhart, a peripheral-initiating event (e.g., acute inflammation and infection) results in changes in the physiology of mechanosensitive, chemosensitive, and/or thermosensitive visceral receptors that might not revert to the baseline, normal state after resolution of the insult. In such a situation, even after the removal of the noxious stimulus, the pain is not suppressed due to changes in the PNS and, particularly, in the CNS that perpetuates visceral hyperalgesia and chronic pain[27].

## The Brain-Gut Axis

The concept of a functional, bidirectional pathway that connects the gastrointestinal tract and the brain and spinal cord (CNS) was originally proposed in the early 1980s and coined as *brain-gut axis*. The brain-gut axis modulates activity of both brain and gut as a result of specific physiological changes[4]. A large neuronal net modulates gut function and reciprocally affects the experience and the regulation of visceral pain. Nerve pathways linking the gastrointestinal tract to the CNS are organized into two parts: the enteric nervous system (the so-called little brain of the gut or intrinsic pathway) and the extrinsic system, which projects along the spinal cord to supratentorial areas of the CNS.

The extrinsic system carries information about the state of the gastrointestinal tract to structures in the CNS (see Fig. 2) and can also result in efferent responses to intestinal organs. This information is conveyed by the vagus nerve (vagal primary afferent neurons have cell bodies in the nodose ganglia) and spinal primary afferent neurons (that have cell bodies in the dorsal root ganglia). The axons of spinal primary afferent neurons in the thoracic and lumbar regions are contained in the splanchnic and mesenteric nerves. The axons of most spinal primary afferent neurons from the colon and rectum with cell bodies in sacral ganglia follow the pelvic nerves. Past research has shown that extrinsic primary afferent neurons react to different types of stimuli, including chemical changes in the intestinal lumen, distension of the gut wall, mechanical distortion of the mucosa, and changes in osmolarity and temperature[28]. This pathway can also be affected by hormones.

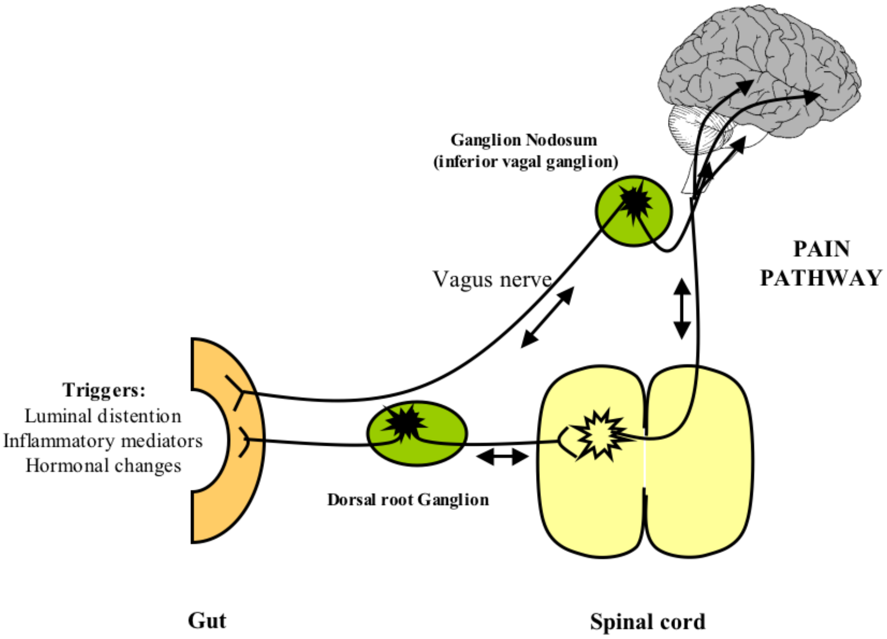

**FIGURE 2**. Diagram showing the extrinsic system of the brain-gut axis.

## The Role of Spinal Cord in Chronic Pain

The spinal cord may play an important role in chronic pain as this structure is not only responsible for the communication between the peripheral system and the brain, but is also responsible for plastic changes that can modulate pain sensation. Visceral sensory axons that are comprised mostly of thinly myelinated A fibers and unmyelinated C fibers constitute 10% of all afferent inflow to the spinal cord. This is a relatively small number when considering the large surface area of some organs.

The dorsal horn of the spinal cord (specifically laminae I, II, V, and X where the sensory fibers terminate) is a critical area for the modulation of sensitivity[29]. Direct tracing studies show that, compared with somatic afferents, the central terminals of visceral afferents spread over more segments in the rostrocaudal dimension, contributing to the vague localization and characterization of visceral stimuli[30]. Both anatomical and electrophysiological studies have demonstrated viscerosomatic convergence in both the dorsal horn and supraspinal centers[31]. This colocalization is responsible for the somatic referral of visceral stimuli. Finally, there is also evidence of viscerovisceral convergence onto these second-order neurons[32]; in other words, same neurons receive information from distinct organs. This evidence together (i.e., the low density of visceral nociceptors, the functional divergence of visceral input within the CNS, and viscerovisceral convergence in the spinal cord) may explain the poor localization of visceral pain[33].

Recently a number of studies have pointed to a possible role of the dorsal column in viscerosensory processing, opening the door for the role of the spinal cord in visceral pain[34,35]. Indeed, a recent review showed that an area of the superficial dorsal horn, the substantia gelatinosa, might undergo plastic changes in the setting of chronic pain[36]. This area receives inputs from sensory afferents that convey noxious sensation and thus can change synaptic connectivity and receptor expression under certain conditions such as following peripheral tissue damage[36]. Not only local modulatory changes, but also the supraspinal structures, can modify the activity of the substantia gelatinosa under certain pathologic conditions and thus affect pain transmission in the spinal cord by activating “top-down” descending facilitatory systems[37].

## The Role of Brain Areas in Chronic Pain

Studies using novel functional brain imaging techniques, such as positron emission tomography (PET) and functional magnetic resonance imaging (fMRI), provided new insights into the central processing of painful stimuli. For instance, these studies have shown that cortical processing of visceral pain may differ from somatic pain as processing of visceral sensations has been localized in the secondary somatosensory cortex (SII) rather than the primary somatosensory cortex that mainly is associated with somatic pain processing.

Studies on the physiology of pain perception have suggested that two different areas in the brain are important for pain representation: (1) the somatosensory cortex (particularly the SII), which receives input from the spinothalamic pathway and is responsible for the sensory-discriminative experience of pain; and (2) the medial thalamus and limbic areas, which are associated with the primary processing of the nociceptive input and responsible for the motivational-affective experience of pain[38,39]. The anterior cingulate cortex (ACC), an important component of the limbic system, receives input from the medial thalamus and other nuclei that receive input from nociceptive neurons, bypassing the somatosensory area. It is therefore considered part of the motivational-affective aspects of pain[2,40,41] and thus may be a useful target for therapies aiming at the pain control.

Several studies have demonstrated the activation of these two areas in the processing of visceral pain. In a magnetoencephalography (MEG) study, Schnitzler et al. showed that visceral afferents project to the SII cortex primarily[42]. In addition, PET and MRI studies have shown activation of inferior SI and frontoparietal operculus areas including SII cortex during the mechanical stimulation of the esophagus[43,44] or rectal stimulation[45]. The lack of visceral representation on SI might account for the poor spatial localization of visceral pain. Other studies have shown that intraesophageal stimulation by acid perfusion is associated with activation of the limbic system in areas adjacent or within the ACC and is similar to the results when pain is elicited by balloon distention, albeit significantly slower, suggesting that the brain may respond differently, depending on type of stimulus. Interestingly, intraesophageal acid perfusion has been shown to evoke cerebral cortical activation prior to the development of heartburn[46].

One of the first investigations of functional abdominal pain examined cerebral activation following painful rectal distension and its anticipation in healthy subjects compared to patients with IBS. Normal subjects show activation of the ACC in response to simulated rectal distension, and symptom intensity and regional activation are strongly correlated in the ACC. IBS patients, on the contrary, do not show increased ACC activity, but an activation of the left prefrontal cortex with anticipation of rectal distension. One potential explanation for the lack of ACC activation in IBS patients is that ACC displays high opiate receptor density, and its activation may include central pain inhibition; therefore these patients may not activate this area and rather activate the frontal lobes that could reflect a vigilance network and maintain a dysfunctional, nonadaptive activity in the neural circuitry involved in pain processing[47]. However, another study showed that patients with IBS had greater thalamic and anterior cingulate activation with painful compared with nonpainful stimuli[48], showing that the precise neural network involved in visceral hyperalgesia is still unclear.

Further studies support the role of SII in chronic abdominal pain in chronic pancreatitis. In this condition, there is evidence of an altered perception of pain that might be sustained by a pancreas-independent mechanism. The fact that videothoracoscopic splanchnicectomy results in limited pain relief in patients with chronic pancreatitis and that total pancreatectomy fails to relieve pain in up to 30% of these patients[49,50] suggest that chronic inflammation of the pancreas could lead to plastic changes in the brain and spinal cord. Our preliminary data (unpublished) from a magnetic resonance spectroscopy study in these patients have shown an imbalance of GABA and glutamate in SII area, with a relative decrease in GABA and an increase in glutamate, indicating an increase in the local cortical excitability.

In summary, visceral pain may lead to plastic changes in the neural network of pain that ultimately are responsible for sustaining pain, regardless of the input from the visceral nociceptors. In such a scenario, new therapeutic approaches should target these dysfunctional areas in the human brain to alleviate CAVP.

## TREATMENT OF CHRONIC ABDOMINAL VISCERAL PAIN

Although a number of pharmacologic and nonpharmacologic therapies are available for patients with chronic pain, these treatments fail in most of the patients. We will discuss briefly these treatment options as well as novel approaches for the treatment of CAVP (see the diagram showing the possible targets for CAVP treatment in Fig. 3).

### Pharmacological Treatment

Although pharmacological treatments are efficacious to relieve acute pain, they do not offer the same benefits for patients with chronic pain. One of the reasons is that the pathophysiology of chronic pain is different from the acute pain. We will discuss the pharmacological options briefly (see the review by Kendrick[51] for a complete description of the available drugs for chronic pain).

### Drugs Affecting the Peripheral Nervous System

NSAIDs are the most-used analgesic drugs. Although this class of drug is effective to alleviate pain, it is associated with several side effects (i.e., interference with platelet aggregation, dyspepsia, gastric ulceration, nephrotoxicity, hepatic toxicity) that preclude the chronic use of these drugs. Furthermore, in many cases, they may fail to relieve chronic pain completely. Perhaps the main reason for this lack of effect is that the effect of these drugs is due to the inhibition of inflammatory mediators, prostaglandins (through the inhibition of COX enzymes). Because chronic visceral pain usually is not associated with injury and inflammation, these drugs might not be suitable for this condition. Therefore, novel drugs with different targets are warranted. Recent pharmacological research has shown promising results in the development of new pharmacological components.

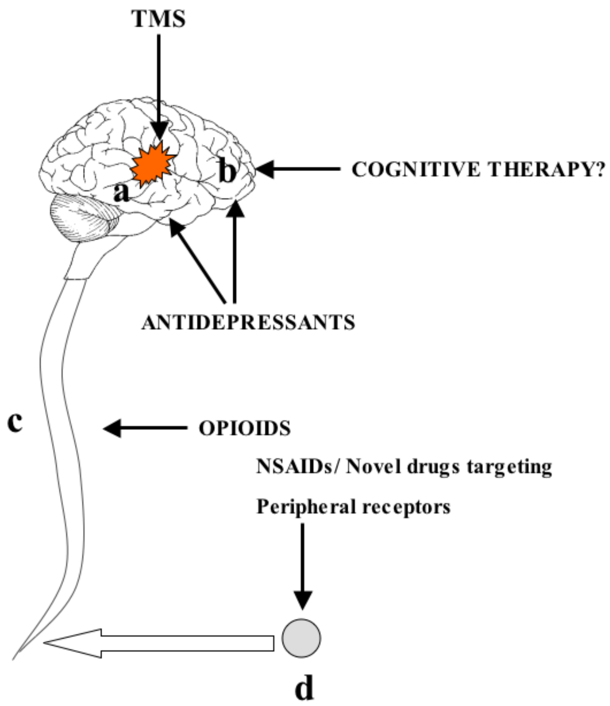

**FIGURE 3**. Examples of treatments used for chronic pain and its potential targets in the nervous system areas (a, secondary somatosensory area; b, limbic areas including the cingulate cortex; c, spinal cord; d, peripheral receptors).

One example is serotonin (5-hydroxytryptamine, 5-HT) that is a key potentiator of gut motility (e.g., stimulates peristalsis), sensation, and secretion. Up to 95% of this neurotransmitter is stored in the enterochromaffin cells, along with neurons, mast cells, and smooth muscle cells located in the gastrointestinal tract. Past research has identified several potential therapeutic receptors such as 5-HT3, 5-HT1A, 5-HT2B, and 5-HT4 that are associated with abdominal pain/discomfort[52]. Some serotonergic agents that are recently available include the 5-HT4 agonist tegaserod (which is approved for constipation-predominant IBS and its effects on visceral sensitivity are, however, less clear[53,54]) and the 5-HT3 antagonist alosetron[55,56]. Randomized clinical trials demonstrate that alosetron improves global IBS symptoms as well other individual symptoms such as abdominal discomfort/pain, stool frequency, stool consistency, and urgency in nonconstipated IBS patients. This drug exerts its beneficial effects on IBS symptoms through central and peripheral action affecting motor and mechanoelastic properties of the gut, water and chloride secretion, and also alters brain activation of limbic areas in the brain that are associated with visceral perception and autonomic circuits[55,56,57,58,59].

Other new serotonergic agents include cilasentron and renzapride. Cilansetron is a novel 5-HT3 antagonist that demonstrated efficacy in males with diarrhea-predominant IBS (IBS-D). Large, randomized, placebo-controlled multicenter trials have shown that this drug is efficacious in the treatment of diarrhea-predominant IBS[60,61]. Renzapride is both a 5-HT3 antagonist and 5-HT4 agonist. This drug is a potentially promising treatment proposed for constipation-predominant IBS that improves stool consistency and increases the frequency of bowel movements. However it has not proved to provide any overall relief from abdominal pain and discomfort[62].

Among the new pharmacological agents studied for the alleviation of visceral pain, kappa-opioid receptor agonists may act, at least in part, by blocking the voltage-dependent sodium channel in vagal and spinal neurons, thereby decreasing neuronal excitability[63,64]. A recent study evaluated the effect of asimadoline (an agonist for kappa-opioid receptors) on colonic compliance, sensory thresholds, and tone in constipation-predominant IBS patients. Asimadoline was associated with a reduced colonic perception in response to balloon distention[64,65,66].

The family of voltage-gated sodium channels might be also a valuable potential target for future therapies. The voltage-gated sodium channels, of which several subtypes are expressed in primary sensory neurons, are essential for action-potential propagation along axons and for the control of membrane excitability. The sodium currents that they mediate are classified electrophysiologically into two types on the basis of their sensitivity to the natural toxin, tetrodotoxin (TTX). All spinal ganglion neurons express TTX-sensitive sodium currents, but TTX-resistant currents seem to be preferentially associated with nociceptive primary afferent neurons. Modulation of the TTX-resistant sodium current has been proposed as a molecular substrate for the sensitization of nociceptors. Indeed, studies using isolated DRG neurons as a model system for the nociceptor terminals have shown that inflammatory mediators such as prostaglandin E2 (PGE2) and serotonin enhance the TTX-resistant current[67,68,69].

Another channel that can underlie the phenomenon of peripheral sensitization and thus contribute to chronic pain is the TRPV1, a nonselective cation channel that is gated by noxious heat (42–53°), protons (pH 5–6) and by various lipid derivatives, including lipoxygenase metabolites of arachadonic acid[70,71]. TRPV1, therefore, may be sensitized by inflammatory mediators that result in a decrease in the threshold for activation and an increase in the size of response for suprathreshold stimuli.

An alternative potential target is the protease receptor-2 (PAR-2). It has been shown that activation of PAR-2 on primary afferent nerves by mast cell–derived tryptase is one of the mechanisms of sensitization in functional bowel disease. Thus, mast cell activation might represent a mechanism for inducing hyperalgesia and PAR-2 antagonists could offer therapeutic benefit[72,73,74].

Moreover, immunohistochemical studies have indicated that calcitonin-gene related peptide (CGRP) is one of the primary neurotransmitters of the spinal afferent neurons[75]. This neurotransmitter is also released by the enteric nervous sensory nerves (IPANs) and its production is stimulated by serotonin. Blockade of CGRP with antagonists, in animals, inhibits visceral pain[76].

Promising results have also been obtained with CRF-1 antagonist. CRF is a key neuroendocrine hormone involved in central stress responsiveness and in the mediation of the stress-related peripheral effects and behavioral responses[77]. Recently, a nonselective CRF receptor antagonist has been demonstrated to reduce significantly abdominal pain and anxiety ratings evoked by electrical stimulation applied in the rectum of IBS patients, but not controls[78].

Another family of neurotransmitters that have a role in visceral pain is the tachykinins (substance P, neurokinin A and B). The effects of tachykinins are mediated by three different receptors — neurokinin 1, 2, and 3 – NK(1), NK(2), and NK(3). Because tachykinins have implications in visceral sensitivity and pain, antagonists of these small peptides might reduce chronic visceral pain. NK1 receptors might modulate intestinal mucosal inflammation. Indeed, an animal study showed that NK1 receptor antagonist CJ-11974 has a weak trend towards increased pressure threshold for discomfort following repetitive sigmoid distension[79]. Moreover, experimental data indicate that NK2 receptor antagonists reduce the hyper-responsiveness that is observed following the application of stressful stimuli in animals[80]. The blockade of peripheral NK2 receptors is considered as a possible mechanism for decreasing the painful symptoms and, indeed, in healthy volunteers, the selective NK2 antagonist nepaductant has proved to reduce the IBS-like symptoms triggered by intravenous infusion of neurokinin A[81].

Finally, although not much data are available, probiotics might play a role in the treatment of CAVP, especially IBS. Probiotic-related improvements include lessening of abdominal pain[82,83]. Among the possible mechanisms of probiotic therapy, the stimulation of host defense systems, such as immune system modulation and the competitive elimination of pathogens, have been suggested as potential explanations[84]. More prospective research with probiotics is needed to study both relief of symptoms and their possible immune-modulating effects.

## Drugs Affecting the Central Nervous System

Opioids are one of the most-used drugs in patients with chronic pain. For instance, the use of morphine in the general population was estimated at 3.5 million grams per 100,000 people in 1996[85]. There are opioid receptors in several areas of the CNS, such as in the spinal cord (dorsal horn), olfactory bulb, nucleus accumben, amygdale, and cortical areas. Thus, this class of drug may have widespread action throughout the CNS. However, this drug is associated with significant adverse events such as somnolence, respiratory depression, decreased bowel motility, and tolerance that can reduce its analgesic effects following chronic use.

Tricyclic antidepressants (TCAs) are a class of drug that has been extensively used in patients with chronic pain. Besides its antidepressant effects that might be advantageous in patients with chronic pain, this class of drug also has independent analgesic effects. The mechanism of its analgesic action is uncertain. It has been speculated that its analgesic properties are related to its action as serotonin and noreprinephrine reuptake inhibitors as well as to a potentiation of endogenous opioids[86,87,88].

Another class of antidepressants that can be effective to relieve chronic pain is the selective serotonin reuptake inhibitors (SSRI). The mechanism of analgesia with SSRI is less well studied and understood compared to that of TCAs and it has been proposed that it may be associated with its primary effect on depression or because of an increase in the serotonin levels.

Other drugs that are commonly used along with antidepressants are the anticonvulsants (such as phenitoin, carbamazepine, valproic acid, clonazepam, and newer agents, gabapentin and lamotrigine). These drugs may be particularly beneficial for chronic pain. The mechanism of action of anticonvulsants in chronic pain is unclear, but their effects on reducing the excitability of the nervous system may play an important role[89,90,91]. Although some benzodiazepines used as antiepileptic drugs are commonly employed in patients with pain, the prolonged use of this drug does not offer benefits for these patients and can lead to the phenomenon of tolerance and dependence. It is interesting to note that antidepressants and anticonvulsants are not associated with a primary analgesic effect (i.e., if these drugs are used in acute pain, there will be no analgesic effect). These drugs, on the contrary, have analgesic effects in chronic pain due to their modulatory effects on the nervous system activity.

## Psychological Interventions

If modulation of the CNS can relieve chronic pain, then one can argue that other forms of CNS modulation can also result in pain amelioration. Indeed, there are a number of studies directly evaluating the effect of psychological interventions on gastrointestinal visceral sensitivity since it has been shown that psychological distress can decrease pain threshold[41,92,93]. A recent meta-analysis of psychological treatments found an overall benefit in reducing pain and other symptoms, such as anxiety and depression, in patients with IBS[94]. Some psychological interventions are related to forms of hypnotherapy. Controlled studies with IBS patients report improvements in abnormal sensory perception with hypnotherapy compared to no treatment or supportive psychotherapy[95,96,97]. Hypnotherapy is effective on both short- and long-term symptom scores in functional dyspepsia as well, with a reduction in medication use and consultation rate[98].

Cognitive behavioral therapy (CBT) has also been investigated for adjuvant treatment of chronic pain. It has been shown that visceral pain is associated with an increased activity in the limbic system and a recent study showed that CBT is associated with changes in the activity of cingulate gyrus[99], an important component of the limbic system. CBT vs. educational therapy was examined in one of the largest studies of psychological treatment in IBS[100]. In the intention-to-treat and per-protocol analyses, CBT shows a significant beneficial response in 70% of the patients vs. 37% of the patients receiving education. The least beneficial effect is observed for IBS patients with depression. A further study compared paroxetine with psychotherapy and routine care in patients with severe IBS, evaluating also the economical impact of such therapies. The results showed that CBT improves health-related quality of life at no additional cost[101].

Further studies are needed to clarify which patients are more likely to respond to psychological treatments and to elucidate the underlying mechanisms for the beneficial effects of these interventions.

## Nonpharmacological Approaches

Patients who fail medical treatments often are referred for nerve blocks or potential surgical resections. However, percutaneous or endoscopic celiac nerve blocks with either alcohol or steroids have had only limited success in chronic pancreatitis. Even among responders, symptoms frequently recur within 2–6 months, albeit the nerve block can be repeated as needed. Furthermore, serious complications have been described associated with this procedure[102,103,104,105].

Surgery is indicated in selected individuals and involves resection usually of a portion of the involved organ. For instance, in chronic pancreatitis, the removal of the pancreas (typically the tail or head) and less commonly the entire pancreas has been advocated as a procedure to reduce pain in these patients. Although resection of the pancreas may provide pain relief in up to 60% of selected patients with chronic pancreatitis, including those with disease extending to the tail of the pancreas[106,107,108], total pancreatectomy should be reserved as a last resort in those who fail all other pain relief treatments. The procedure can be debilitating, leading to exocrine and endocrine dysfunction. Interestingly, even with the complete removal of the gland, pancreatic pain persists in up to 30% of patients[49,109], suggesting secondary dysfunction of the CNS.

Despite the fact that surgical procedures can provide some benefit for patients with chronic visceral pain, they are associated with adverse effects and frequent treatment failure. The main reason for lack of efficacy is the fact that chronic visceral pain syndromes may result in CNS changes that cannot be reverted by a change in the peripheral afferent system. Therefore, new approaches should target brain cortical areas, rather than the PNS, to relieve pain in patients with chronic visceral pain.

## Possible Future Therapeutic Approaches: Brain Stimulation for the Treatment of Visceral Pain

It has been shown that alterations in the SII activity is associated with refractory abdominal visceral pain. Pilot data (not published) from our laboratory have shown that patients with chronic pain due to chronic pancreatitis have a local increase in the cortical excitability of SII due to an imbalance of increased glutamatergic and decreased GABAergic activity. If SII hyperexcitability plays a role in pain generation, a focal decrease in brain excitability may be helpful in pain control. One candidate tool to modulate brain activity is repetitive transcranial magnetic stimulation (rTMS).

TMS is a form of brain stimulation that is based on a time-varying magnetic field. A small coil with a powerful and rapidly alternating electrical current is applied over the brain cortex (Fig. 4). The continuous change in the electrical current that flows through the coil can generate a time-varying magnetic field that passes unimpeded through skin and bones, generating an electric current inside the skull, where it can be focused and restricted to small areas depending on the coil geometry and shape[110]. It has been demonstrated that this current, if applied repetitively (rTMS), might induce a cortical modulation that lasts beyond the time of stimulation.

Several animal and human studies establish that rTMS can modulate brain cortex activity noninvasively[110,111,112,113,114,115]. The modulation may range from suppression to facilitation of activity in the targeted cortical area, depending on the stimulation parameters (particularly frequency of stimulation)[114,116,117]. While low-frequency (1 Hz) rTMS can decrease the excitability of targeted cortical regions resulting in a measurable behavioral changes, high-frequency (20 Hz) rTMS often has the opposite effects[114,115,117,118,119,120,121]. Furthermore, several animal studies report that rTMS is associated with modulation of NMDA binding sites[122] and can increase immediate-early gene expression[123].

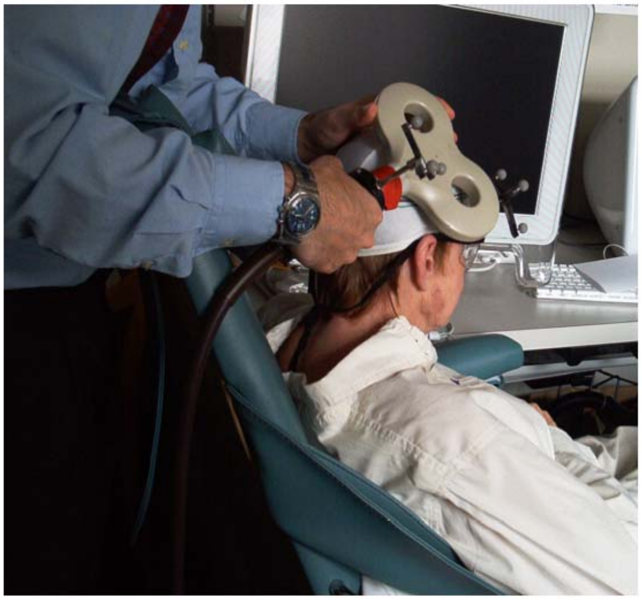

**FIGURE 4.** Picture showing the application of rTMS treatment; the rTMS coil is held over the subject's scalp.

Clinically, several studies have shown that the cortical activity modulation by rTMS can be successfully used in the treatment of various neuropsychiatric disorders[124,125,126,127,128], as well as the treatment of chronic pain[129,130,131,132]. The notion that brain modulation may be an effective therapy for chronic pain syndromes is based on the fact that chronic electrical epidural stimulation of the precentral cortex can improve drug-resistant neurogenic pain[133,134]. Presumably this is due to inhibition of nociceptive neurons at cortical levels through non-noxious fibers from the motor cortex. However, this technique is invasive and requires a craniotomy. Two recently published studies using rTMS applied to the motor cortex reported that this treatment resulted in less pain in patients with therapy-resistant chronic somatic pain syndromes[130,135]. In addition, Lefaucher et al. studied 60 patients suffering from intractable pain. These patients underwent one session of active or sham rTMS in random order. The authors showed that pain reduction was significantly greater following real than sham rTMS[129].

Because of these promissory results of brain stimulation for the treatment of chronic pain, a recent study has investigated whether rTMS is effective in the relief of chronic visceral pain in patients with chronic pancreatitis[136]. The effect of low- and high-frequency rTMS was studied. Five participants with idiopathic chronic pancreatitis underwent (in random order) six sessions of rTMS of either the right and left SII with different parameters of stimulation: right 1 Hz-rTMS (R-1Hz), left 1 Hz-rTMS (L-1Hz), right sham (R-sham), left sham (L-sham), right 20 Hz-rTMS (R-20Hz), and left 20 Hz rTMS (L-20Hz). Each participant had chronic daily pain for at least 3 years. Baseline pain scores on a visual analogue scale were obtained during the 2 weeks prior to treatment. Pain levels were assessed daily throughout the TMS sessions. Daily chronic and "as needed" (prn) analgesic intake was also recorded. The interval between each session was 1 week to avoid carryover effects. Participants and raters were blinded to the stimulation condition.

Three out of five participants had a significant response with a mean pain reduction of 59% after rTMS to the right SII at 1-Hz frequency (R-1Hz), as compared to baseline. This improvement lasted 3–5 days. Other conditions including sham treatments either had no effect or were associated with a worsening of the pain score. When the results were analyzed together, only R-1Hz resulted in a consistent improvement in pain across participants.

In summary these preliminary data suggest that brain stimulation, in particular rTMS, is effective to treat chronic somatic and visceral pain. Furthermore, as repeated sessions of rTMS can increase the clinical benefits of this therapy and does not increase the risk to subjects, it should be further tested in patients with CAVP as well as stimulation of other sites, such as the primary motor cortex that is effective in relieving somatic pain.

## FUTURE DIRECTIONS

Although several studies have investigated the pathophysiology of CAVP, the mechanisms underlying this disorder are not yet fully elucidated. Furthermore, most of the available therapies to relieve CAVP are not effective. Herein we proposed that one of the mechanisms of chronic pain is plastic cortical changes and thus, a therapy focusing on the modulation of this cortical dysfunctional activity would be desirable. The data suggest that rTMS is a treatment that can modulate cortical excitability in a painless and noninvasive way and can be effective in the relief of chronic pain in patients with chronic pancreatitis.

If rTMS can be effective to relieve CAVP, other neuromodulatory approaches might also result in the same therapeutic effect. For instance, invasive brain stimulation, such as deep brain or epidural cortical stimulation, can be successfully used to treat neuropsychiatric disorders, such as depression[137], epilepsy[138], and, indeed, somatic and central pain[134,139]. Therefore, similar to rTMS, it could have a positive impact on visceral pain as well. Moreover, a further, less-invasive technique of brain stimulation — vagus nerve stimulation (VNS) — has been reported to have antidepressant effects[140] and perhaps might have some analgesic effects in patients with CAVP. Finally, another type of noninvasive brain stimulation — transcranial direct current stimulation — is being explored as a treatment for epilepsy and depression. Preliminary data have shown positive results of this technique for depression[141] and epilepsy[142]. Furthermore, in a preliminary study, we showed that this technique is associated with a significant analgesic effect in patients with central pain due to spinal cord injury[143]. This device has the advantage of being simpler and cheaper than rTMS and perhaps can be designed as a portable device in the future.

Other targets in the CNS, rather than cortical areas, may be important in pain relief in CAVP. For instance, a recent study has shown that spinal cord stimulation (SCS) is an efficacious technique for the treatment of visceral pain syndromes. In this study, there was a substantial overall mean reduction in the Visual Analog Scale (VAS) pain scores as well as a decrease in narcotic use with minimal complications. Spinal cord stimulation might be an effective, relatively noninvasive and reversible procedure for visceral chronic pain, but the clinical utility of this approach needs to be further evaluated[144].

Individualized parameters of brain stimulation may be an important factor predicting response and needs further study. Parameters of the activity of the stimulated area should guide the necessary dosage of brain stimulation. For instance, online electroencephalography (EEG) could be used to monitor the effects of rTMS and adjust the doses of this treatment according to its impact on the cortical excitability. Not only EEG, but also neuroimaging techniques, could be used as a method of adjusting the TMS parameters.

Although we acknowledge that it is too early to make strong predictions about the role of brain stimulation in the clinical management of patients with CAVP, the current data make us optimistic about the clinical utility of this new therapeutic tool in visceral pain, indicating, therefore, that this is a topic that deserves to be further studied.

## ACKNOWLEDGMENTS

This work was supported by a grant from the Harvard Medical School Scholars in Clinical Science Program (NIH K30 HL04095) to F.F. and by grants from NIH (DK071851-01) and The National Pancreas Foundation to S.D.F., A.P-L. and F.F.

## BIOSKETCHES

**Franca Patrizi, MD,** was a Visiting Fellow in the Pancreas Center at Beth Israel Deaconess Medical Center and Harvard Medical School, Boston. Currently, she is a Clinical Gastroenterology Fellow at University of Verona, Italy.

**Steven D. Freedman, MD, PhD,** is Director of the Pancreas Center at Beth Israel Deaconess Medical Center. He is also Chief of the Division of Translational Research, Director of the NIH-funded General Clinical Research Center, and an Associate Professor of Medicine at Harvard Medical School, Boston. Dr. Freedman's expertise is in exocrine pancreatic disease with a particular focus on pancreatitis and cystic fibrosis in both adults and children. He has an extensive research program that encompasses both basic science as well as clinical trials of new therapies for pancreatitis and cystic fibrosis.

**Alvaro Pascual-Leone, MD, PhD**, is the director of the Center for Non-Invasive Brain Stimulation and Professor of Neurology at Harvard Medical School and the Beth Israel Deaconess Medical Center (Boston). He is also the Associate Director of the Harvard-Thorndike General Clinical Research Center. Dr. Pascual-Leone’s research focus is to understand neural plasticity at system's level. His focus is to learn enough about the mechanisms of plasticity in order to manipulate them, suppressing some changes and enhancing others, to gain a clinical benefit and behavioral advantage for a given individual.

**Felipe Fregni, MD, PhD,** is an Instructor in Neurology at Harvard Medical School and the Beth Israel Deaconess Medical Center (Boston). Dr. Fregni’s main interest is to develop new treatments for neuropsychiatric diseases, such as chronic pain, using noninvasive brain stimulation tools such as transcranial magnetic stimulation (TMS) and transcranial direct current stimulation (tDCS).
